# An SOI CMOS-Based Multi-Sensor MEMS Chip for Fluidic Applications †

**DOI:** 10.3390/s16111608

**Published:** 2016-11-04

**Authors:** Mohtashim Mansoor, Ibraheem Haneef, Suhail Akhtar, Muhammad Aftab Rafiq, Andrea De Luca, Syed Zeeshan Ali, Florin Udrea

**Affiliations:** 1 Institute of Avionics and Aeronautics, Air University, E-9, Islamabad 44000, Pakistan; ibraheem.haneef@gmail.com; 2 Department of Engineering, University of Cambridge, 9-JJ Thomson Avenue, Cambridge CB3 0FA, UK; ad597@cam.ac.uk (A.D.L.); fu@eng.cam.ac.uk (F.U.); 3 National University of Sciences & Technology (NUST), H-12, Islamabad 44000, Pakistan; sakhtarjanjua@gmail.com; 4 Pakistan Institute of Engineering and Applied Sciences, Nilore, Islamabad 45650, Pakistan; aftabrafiq@pieas.edu.pk; 5 Cambridge CMOS Sensors Ltd., Deanland House, 160-Cowley Road, Cambridge CB4 0DL, UK; zeeshan.ali@ccmoss.com

**Keywords:** SOI, CMOS, MEMS, aerospace, fluid dynamics, dense sensor packing, thermodiodes, DRIE, multi-sensor, sensor system, thermal flow rate sensor, piezoresistive pressure sensor

## Abstract

An SOI CMOS multi-sensor MEMS chip, which can simultaneously measure temperature, pressure and flow rate, has been reported. The multi-sensor chip has been designed keeping in view the requirements of researchers interested in experimental fluid dynamics. The chip contains ten thermodiodes (temperature sensors), a piezoresistive-type pressure sensor and nine hot film-based flow rate sensors fabricated within the oxide layer of the SOI wafers. The silicon dioxide layers with embedded sensors are relieved from the substrate as membranes with the help of a single DRIE step after chip fabrication from a commercial CMOS foundry. Very dense sensor packing per unit area of the chip has been enabled by using technologies/processes like SOI, CMOS and DRIE. Independent apparatuses were used for the characterization of each sensor. With a drive current of 10 µA–0.1 µA, the thermodiodes exhibited sensitivities of 1.41 mV/°C–1.79 mV/°C in the range 20–300 °C. The sensitivity of the pressure sensor was 0.0686 mV/(V_excit_ kPa) with a non-linearity of 0.25% between 0 and 69 kPa above ambient pressure. Packaged in a micro-channel, the flow rate sensor has a linearized sensitivity of 17.3 mV/(L/min)^−0.1^ in the tested range of 0–4.7 L/min. The multi-sensor chip can be used for simultaneous measurement of fluid pressure, temperature and flow rate in fluidic experiments and aerospace/automotive/biomedical/process industries.

## 1. Introduction

The last two decades have witnessed a tremendous growth in the MEMS (Micro-Electro-Mechanical-Systems) market [1]. Compared to their conventional counterparts, the MEMS sensors offer advantages of miniaturization, low power consumption and cost reduction. Furthermore, they also bring in the possibility of multiple sensors and electronic circuit integration on a single chip.

There are quite a few examples reported in the literature [2,3,4,5,6,7,8,9,10,11,12], where multiple sensors have been integrated on a single MEMS chip. Generally, the need to cluster more than one sensor on a chip arises when the investigators are designing a sensor system intended for a specific industrial/scientific application. For example, an array of five pressure, temperature and wall shear stress sensors was integrated on a single chip by Xu et al. for simultaneous measurements of all three parameters in micro-channels [2,5]. Similarly, Kimura et al. [13] arranged hot film sensors in a grid for two-dimensional wall shear stress imaging. Berns and Obermeier reported Aero-MEMS sensor arrays for time-resolved measurement of pressure and wall shear stress [14]. Fabrication of a sensor system using the Complementary Metal Oxide Semiconductor (CMOS) MEMS approach has been reported by Hagleitner et al. [3] with calorimetric, capacitive and temperature sensors accompanied by the required circuits for drive and signal conditioning on the same chip. It is worth mentioning that electronic circuit integration on a single chip, as demonstrated in [3], is possible using the CMOS MEMS approach and is not valid, in general, for all MEMS-based sensors.

In addition to the previous examples, sensor systems designed for laboratory and medical applications sensing temperature, fluid density and mass flow rate [4] and fish behaviour and migration movements monitoring light, pressure, temperature and conductivity [11] were also reported. Simultaneous utilization of multiple sensors for monitoring flow velocity, direction and ambient temperature on a single chip has also been demonstrated by Ma et al. [6]. Roozeboom et al. reported acceleration, magnetic field, wind direction, wind speed, pressure, light intensity, humidity and temperature sensors on a single silicon die [7]. Integrated flow, pressure and temperature sensing capabilities on a single silicon chip have also been reported in [9]. A CMOS chip has also been demonstrated as a pressure-flow sensor by Li et al. [8]. Silicon-On-Insulator (SOI) technology has also been used in an integrated pressure-flow sensor for correlation measurements in turbulent gas flow [10]. Similarly, Yoon and Wise reported a mass flow sensor with an on-chip CMOS interface circuitry [15]. In yet another interesting work, an SOI MEMS chip containing an accelerometer along with pressure, temperature and humidity sensors has been demonstrated by Fujita et al. [16]. This chip was then wire bonded and packaged with another chip produced in a semi-custom Bi-CMOS process containing the sensors’ interface circuitry. A similar System-In-Package (SIP) arrangement has also been reported in [17], where a silicon-on-glass sensor chip capable of sensing temperature, pressure and humidity has been packaged with another Bi-CMOS process-produced independent circuitry chip. For a quick over-view, a brief summary of key multi-sensor MEMS chips reported in the literature since 1987 is presented in Table 1.

In contrast to all these attempts where the benefits of SOI and CMOS technology have not been simultaneously exploited in a single multi-sensor platform, in this work, we report a multi-sensor SOI CMOS MEMS chip that can simultaneously measure pressure, temperature and flow rate in gaseous and liquid flows. The capability to measure flow rate, pressure and temperature simultaneously makes such a chip very attractive for different applications in aerospace, automotive, biomedical and process industries. The incorporated SOI technology brings in the advantages of lower power consumption (since the heating elements are fully embedded within thin dielectric layers for enhanced thermal isolation) and operations at elevated temperatures (up to 300 °C, due to the employment of high quality thin single crystal silicon resulting in reduced junction areas and, thus, lower leakage currents and minimization of latch-up-related issues). Use of a CMOS process for sensor chip fabrication helps in attaining inter-chip/wafer performance reproducibility and provides the benefits of the integration of circuitry on the same chip. Furthermore, in our work, the use of Deep Reactive Ion Etching (DRIE) for the realization of sensors’ membranes results in denser sensor device packing, due to near-vertical membrane cavity walls, in comparison to other wet etching techniques, which would result in slanting walls.

Multiple sensors of similar design were integrated on the same chip keeping in view a number of considerations, e.g., an array of temperature sensors can be utilized for 2D thermal mapping of a hot surface as reported in [19]. Arrays of thermal flow sensors can be used to study the distribution of wall shear stress exerted on the surface of a micro-channel and compared with analytical results [2,5,13]. All three types of sensors, i.e., pressure, temperature and flow rate, are key parameters for experimental fluid dynamics studies. Having multiple sensors on the same chip can help ensure the verification of the individual sensor calibration. Furthermore, the redundant sensors on the same chip can be very useful in industry, where the breakdown of individual sensors results in the complete halt of the assembly line/manufacturing process.

Having given the background and relevance of our work in Section 1, the paper briefly describes the design of the multi-sensor MEMS chip in Section 2. Section 3 is dedicated to the details of the experimental setups for the characterization of individual sensor types. In Section 4, the experimental results are presented and discussed. Conclusions are finally presented in Section 5.

## 2. Multi-Sensor Chip Design

**Figure 1:**
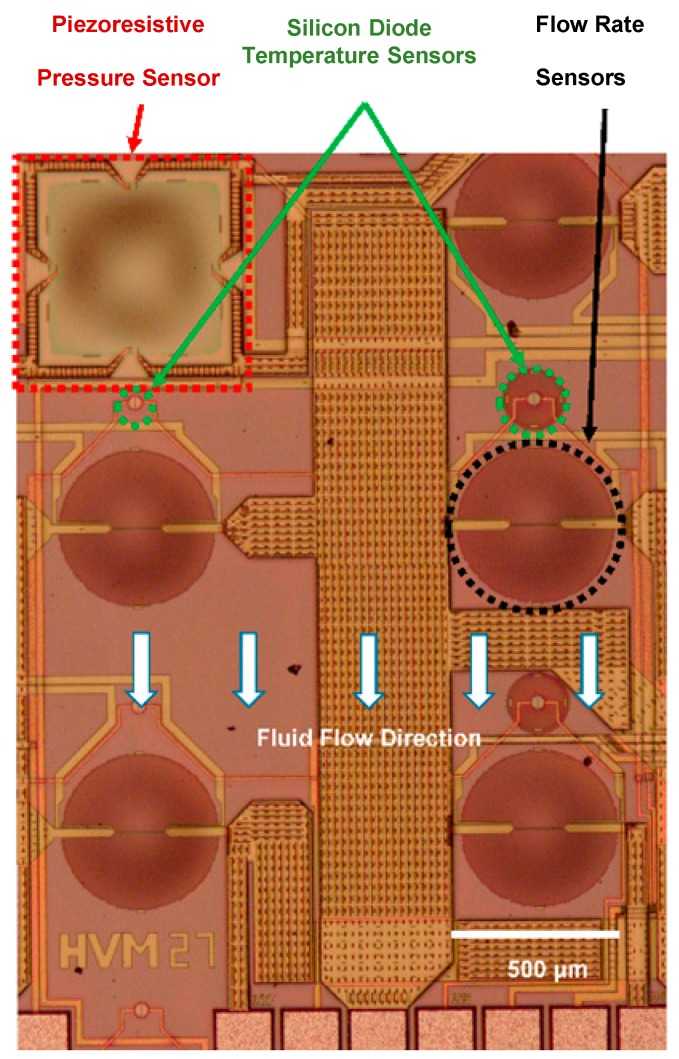
Partial view of the SOI CMOS multi-sensor MEMS chip capable of measuring fluid pressure, temperature and flow rate.

**Figure 2:**
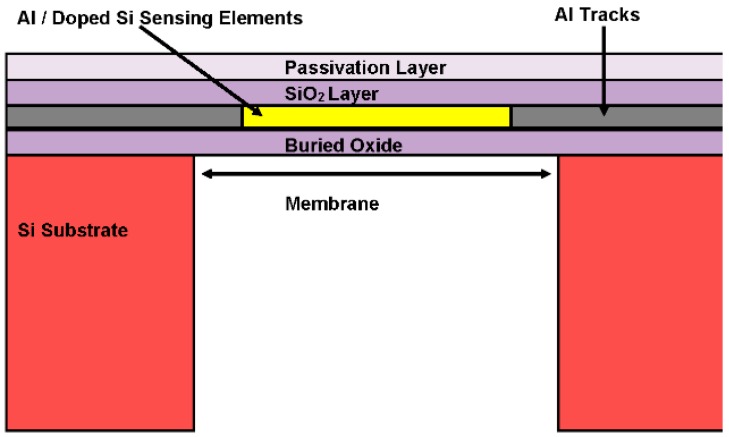
Design of thin silicon oxide membranes realized through DRIE in the SOI CMOS multi-sensor MEMS chip. The Buried Oxide Layer (BOX) layer acts as an effective etch stop for the DRIE process.

Our 3.8 mm × 3.8 mm multi-sensor MEMS chip (Figure 1) is capable of sensing fluid pressure, temperature and flow rate using a piezoresistive diaphragm pressure sensor, ten thermodiodes and nine hot film sensors, respectively. The chip has been designed using Cadence Virtuoso Layout Editor and fabricated in a commercial CMOS foundry. The sensing elements have been embedded in thin silicon dioxide membranes (thickness ~5 µm). The membranes have been realized by a single, post-CMOS DRIE back-etch step in the same foundry. The BOX (Buried Oxide Layer) acts as an effective etch stop during the DRIE step (see Figure 2). Due to the near-vertical walls etched by DRIE, compact packing of devices on the chip has been made possible. Oxide membranes thermally isolate the sensing elements from the substrate, thus allowing the flow sensors’ high electro-thermal transduction efficiency. Details of each type of sensor with a brief theoretical background are given in subsequent paragraphs.

### 2.1. Temperature Sensor

Two broad classes of temperature sensors are absolute temperature sensors and the relative temperature sensors. In absolute temperature sensors, the measured temperature is referenced to absolute zero or some other temperature scale, like Celsius or Fahrenheit. Examples of absolute temperature sensors include thermistors, RTDs (Resistance Temperature Detectors) and thermodiodes. Relative temperature sensors, on the other hand, measure the temperature difference between two points, considering one to be the reference. Thermocouples are one of the most commonly-used examples of relative temperature sensors.

Thermistors and RTDs utilize the predictable change in the resistance of materials with temperature. The resistance change is related to temperature variations by calibration. The material used in RTDs is generally metal, whereas thermistors are generally made of polymers, ceramics or semiconductors. Thermocouples, on the other hand, require two dissimilar materials joined together to form a cold (reference) junction and a hot (sensing) junction. Other types of temperature sensors include optical, acoustic, thermodiode and piezoelectric temperature sensors. Various temperature measurement techniques have been reviewed in [20]. Among all the sensors described above, thermodiodes are one of the most commonly-used temperature sensors. This is mainly because they produce linear output over a wide temperature range [21,22,23], have low power consumption, a small size and high reliability. Most importantly, they can be easily integrated with ICs (integrated circuits) and other sensors.

Thermodiodes are simple temperature-sensitive p-n junctions. Harris [24] reported their first utilization as temperature sensors in 1961. Two common operation modes of thermodiodes are constant current mode and constant voltage mode. Constant voltage mode is implemented by applying a fixed voltage across the diode and results in an inverse relation between the log of current (*I*) passing through the diode and the temperature [25]:

(1) log(I) ∝ 1 ⁄ T

where *T* and *I* are temperature and current, respectively. On the other hand, the constant current mode is implemented by supplying a fixed forward current to the thermodiode. In such a case, the voltage drop across the diode *V*_(*const I*)_ has a ddirectly proportional relation with the temperature.

(2) $$V_{\left(constant\;I\right)}\;\propto \;T$$

Udrea et al. [26] have given comprehensive mathematical details of constant current operation mode in thermodiodes. Similarly, Mansoor et al. [27] have reviewed various applications where silicon diodes have been used for temperature sensing.

**Figure 3:**
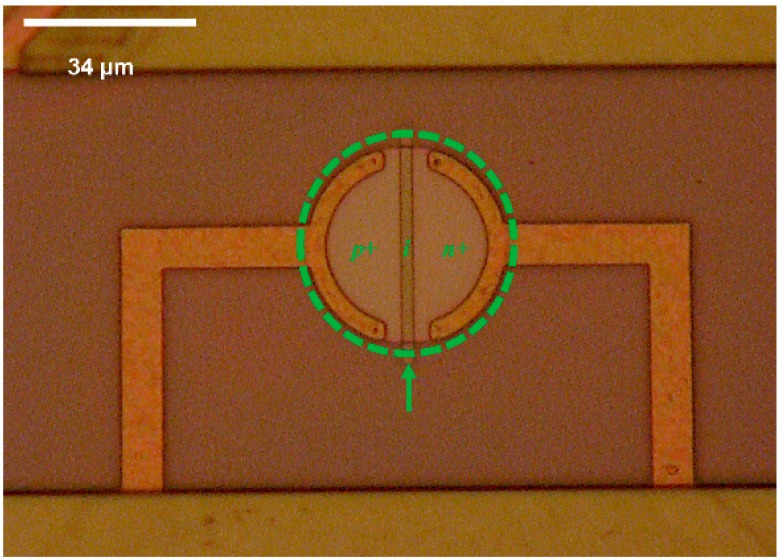
Micrograph of a single thermodiode used as a temperature sensor on the multi-sensor SOI CMOS MEMS chip.

The SOI CMOS multi-sensor MEMS chip contains ten thermodiodes. A micrograph showing a close-up of one of the thermodiodes is given in Figure 3.

### 2.2. Pressure Sensor

Conventional pressure sensors have different types and transduction mechanisms. Manometers, aneroid barometers, bourdon tubes, vacuum sensors and diaphragm pressure sensors are some of the most common examples. However, in MEMS, deflectable diaphragm-type pressure sensors have been reported quite frequently. In such sensors, the applied pressure causes the diaphragm to deflect. The pressure is evaluated by sensing the deflection in the diaphragm. To sense diaphragm deflection, two common techniques are utilized. In one of the techniques, the diaphragm acts as one of the plates of a capacitor. A deflected diaphragm causes the capacitance to change, thus enabling the pressure to be estimated. The other technique uses piezoresistors embedded within the diaphragm to sense the deflection and hence the applied pressure. Interested readers may refer to an excellent review on MEMS pressure sensors by Eaton et al. [28].

**Figure 4:**
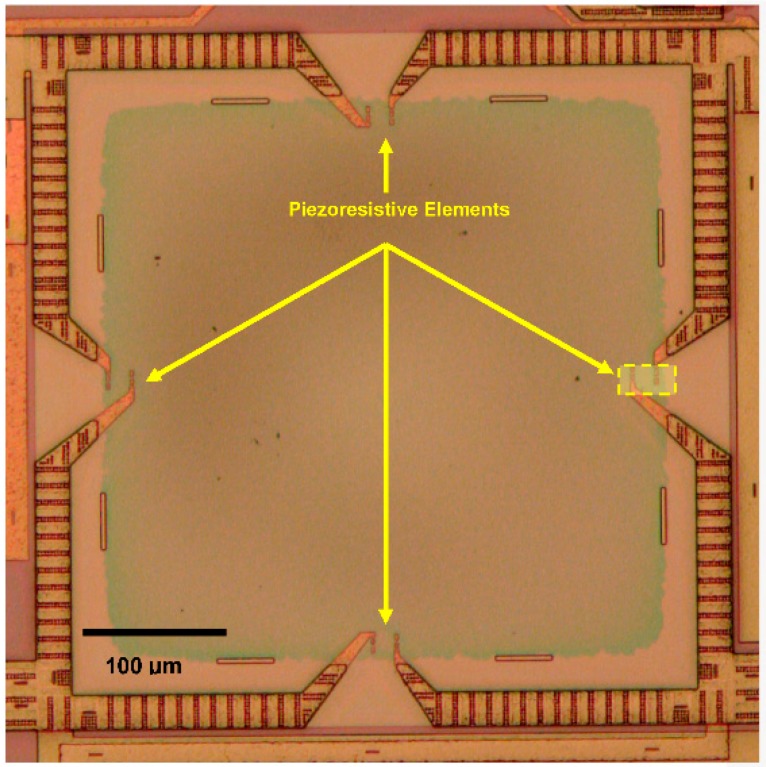
Square membrane pressure sensor in the multi-sensor SOI CMOS MEMS chip. The membrane is made of SiO_2_ and has a side length of 400 µm. The cadence layout of the boxed piezoresistor is shown in Figure 6.

**Figure 5:**
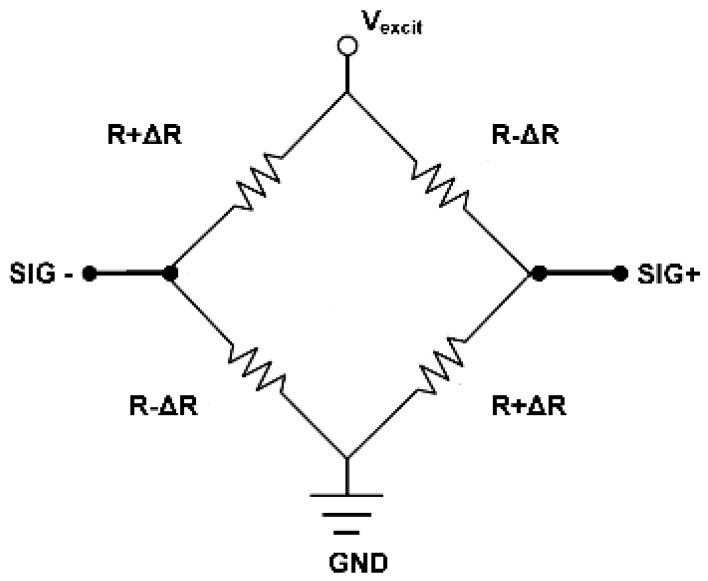
Four piezoresistive elements connected together in a Wheatstone bridge configuration.

**Figure 6:**
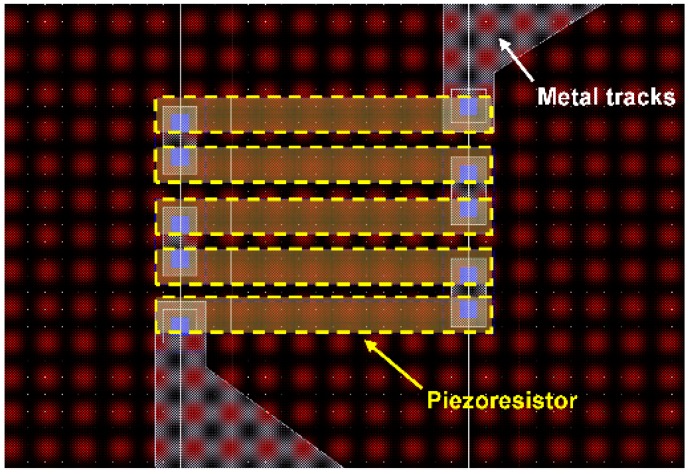
Cadence layout of a meander piezoresistor embedded in the pressure sensor membrane. The piezoresistor has five segments (with each segment 16 μm long and 2 μm wide) joined together by metal contacts.

The pressure sensor in our multi-sensor system has a square diaphragm (Figure 4). When pressure is applied, maximum stresses occur at the middle of the four edges of the square diaphragm [29]. Four *p+*-doped silicon piezoresistors have been embedded at the location of maximum stress in the SiO_2_ membrane in a Wheatstone bridge configuration (Figure 5). All piezoresistors have similar resistance values (~500 Ω). The Cadence layout of a meander piezoresistor embedded in the pressure sensor diaphragm/membrane is shown in Figure 6. Each piezoresistor has five segments while each of these segments is 16 µm long and 2 µm wide.

The bottom side of the pressure sensor was sealed at atmospheric pressure using an adhesive. Upon application of pressure, opposite piezoresistors change their resistance by +Δ*R* and −Δ*R*. As a result, the signal output at *SIG*+ and *SIG*− is offset due to the change in the resistive Wheatstone bridge. The output signal for measurement is given by *V_sig_*:

(3) $$V_{sig}=\left(SIG+\right)-\left(SIG-\right)$$

The change in output signal is calibrated to indicate applied pressure.

### 2.3. Flow Sensor

Van Putten and Middelhoek [30] were the first ones to report a silicon flow sensor in 1974. For a review of flow sensors, the interested readers may refer Nguyen [31]. Thermal flow sensors are based on the principle of heat transfer between the sensor itself and the flowing media. A wire or film is heated by flowing current through it (Joule heating effect). The change in film temperature results in the film resistance change. When the fluid flows over the element, some of the heat is transferred to the fluid, resulting in cooling of the film and, thus, a change in its resistance. In addition to convection losses, conduction and radiation losses are also present. However, heat lost due to radiation is minimized by the top silicon nitride passivation layer that has an emission coefficient lower than 0.2 [32]. Furthermore, the small surface area and relatively low operating temperature are also good reasons to neglect the radiation contribution; whereas the conduction losses from the hot film to the substrate are minimized by embedding the aluminium film in the thin silicon dioxide membrane. As a result, most of the power due to Joule heating is convected to the fluid passing over the hot film [33]:

(4) $$P_{J}=\left(G_{cond}+G_{conv}+G_{rad}\right)\left(T-T_{amb}\right)$$

Or

(5) $$P_{J}\approx G_{conv}\left(T-T_{amb}\right)$$

where $\left(P_{J}=Current\;\times Voltage\right)$ is the power consumption and $G_{cond},G_{conv}\;\mathrm{and}\;G_{rad}$ are thermal conductance due to conduction, convection and radiation, respectively. $\left(T-T_{amb}\right)$ is the rise of the temperature of the hot film. Thermal conduction due to convection $G_{conv}$ is given by:

(6) $$G_{conv}\approx h2S_{M}$$

Here, h is the convective heat transfer coefficient and $S_{M}$ is the area of the membrane. In the case of forced convection, h is proportional to $v^{n}$ [32], where v is the velocity of fluid and n depends on the flow regime. Thus, the heat transferred from the hot wire/film to the fluid is measured by the change in its resistance and calibrated to represent the flow rate of the fluid.

**Figure 7:**
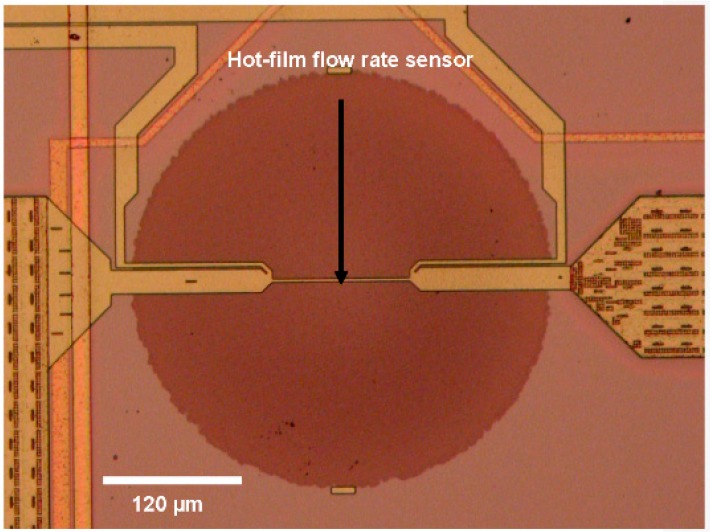
Close up view of hot film flow rate sensor on the multi-sensor SOI CMOS MEMS chip. The aluminium-based sensor hot film is 120 µm long and 2 µm wide. The diameter of the silicon oxide membrane is 360 µm.

The multi-sensor MEMS chip contains nine embedded flow rate sensors. The aluminium-based sensing elements are 120 µm long and 2 µm wide. The device is back etched by DRIE to leave the hot film element suspended on a thin silicon dioxide membrane (Figure 7). Though the hot film sensors have been used for flow rate sensing, the same sensors can be used for measuring wall shear stress sensors, as well [2,5].

## 3. Calibration Setups

### 3.1. Temperature Sensors

A probe station with an integrated temperature controlled hot chuck and a Keithley 2400 Source Meter Unit (SMU) was used to characterize the temperature sensors in constant current mode. The setup can provide thermal characterization within an accuracy of 1 °C. To ensure thermal stabilization, the apparatus was maintained at the required temperature conditions for the requisite time (5–7 min).

### 3.2. Pressure Sensor

**Figure 8:**
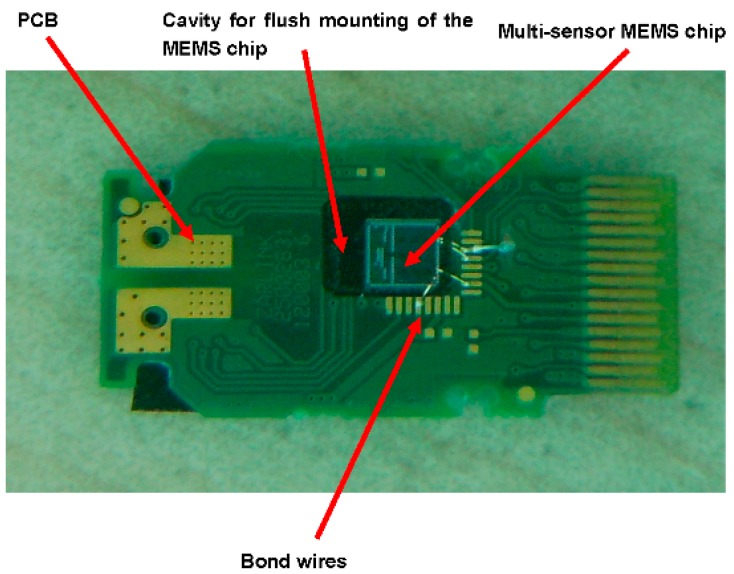
Multi-sensor MEMS chip bonded on a PCB. A cavity was carved in the PCB to accommodate the MEMS chip. The rest of the cavity was filled up with a UV-cured adhesive that ensured stress-free sealing of the chip and the pressure sensor.

For pressure characterization, the multi-sensor MEMS chip was packaged on a custom-designed PCB. An Ultra Violet (UV) adhesive (Norland Optical, Cranbury, NJ, USA, Adhesive 61 commonly known as “NOA 61”) was used to seal the chip in the PCB as shown in Figure 8. The NI (National Instruments, Austin, TX, USA) PXI-4130 SMU and the 4070 Digital Multi-Meter (DMM) were used to acquire electrical signals. The packaged chip was placed in a sealed chamber with separate ports for pressurizing the chamber and measuring the chamber pressure with the help of the DH-Budenberg-365 Pressure Calibrator (Manchester, UK).

### 3.3. Flow Rate Sensor

The MEMS chip was packaged in a CPGA (Ceramic Pin Grid Array) for characterizing flow rate sensors. A smooth surface was provided to airflow over the sensors by filling gaps around the chip with the help of UV adhesive.

**Figure 9:**
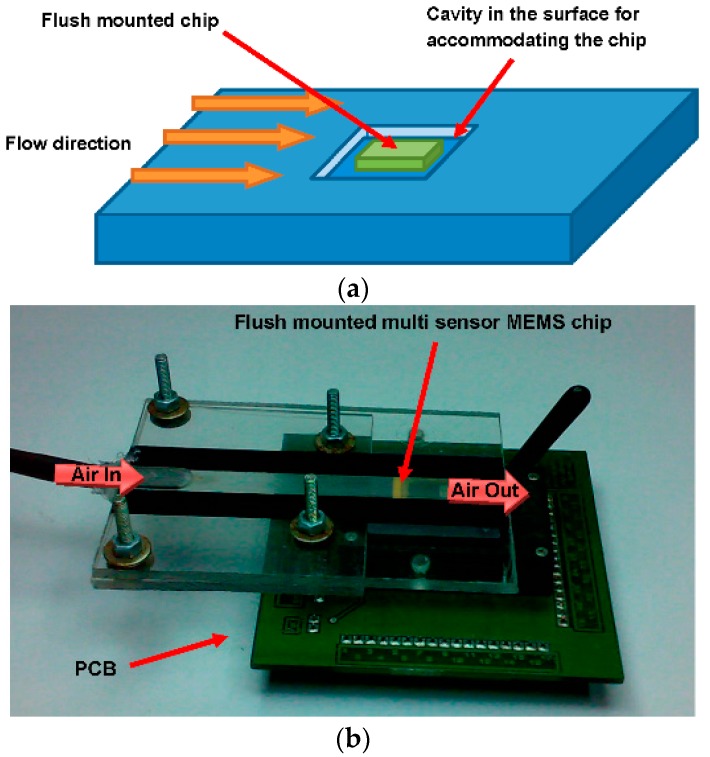
(**a**) Schematic diagram of the packaged multi-sensor MEMS chip for simultaneous measurement of pressure, temperature and flow rate; (**b**) multi-sensor MEMS chip packaged in a micro-channel for flow rate characterization. After packaging of the chip, the space left around the chip was filled with a polymer adhesive to ensure smooth flow over the chip.

An adhesive was used to fill the gap between the sensor and Ceramic Pin Grid Array (CPGA) walls and to provide a smooth surface to air flow. The schematic of the multi-sensor MEMS chip in a potential application is shown in Figure 9a. The chip has been designed for applications requiring simultaneous measurement of temperature, pressure and flow rate/wall shear stress. Potential applications include instrumentation in wind tunnel testing, microfluidics and real time monitoring of parameters on aerodynamic surfaces in real time. The packaged sensor was flush mounted in a Plexiglas micro-channel for characterization (Figure 9b). The channel had a length of 47 mm with a rectangular cross-section that was 500 µm high and 6 mm wide.

## 4. Results and Discussion

Independent experimental setups were used to characterize all the three types of sensors embedded in the multi-sensor SOI CMOS MEMS chip. Detailed results for each type of sensor have been presented and discussed in subsequent paragraphs.

### 4.1. Temperature Sensors

**Figure 10:**
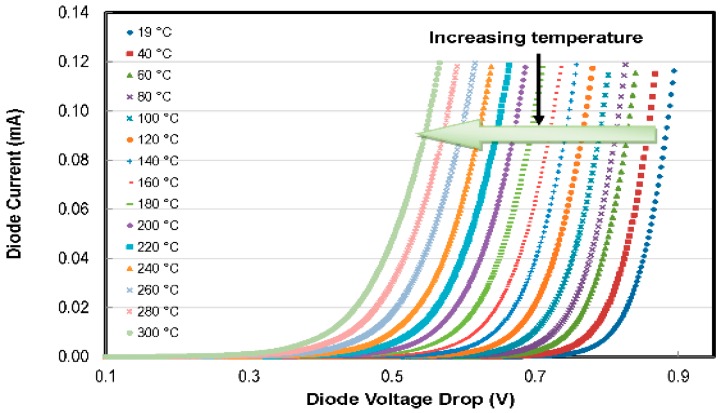
Shifting of the *I-V* curve of a thermodiode temperature sensor with increasing temperature.

To verify the reproducibility of the results among different MEMS chips, thermodiodes from different chips have been characterized, and the results have been compared. Characteristic current-voltage (*I-V*) curves of the diodes have been recorded over the temperature range of 20 °C–300 °C. Effects of changing temperature on the I-V characteristics of the thermodiode are given in Figure 10.

**Figure 11:**
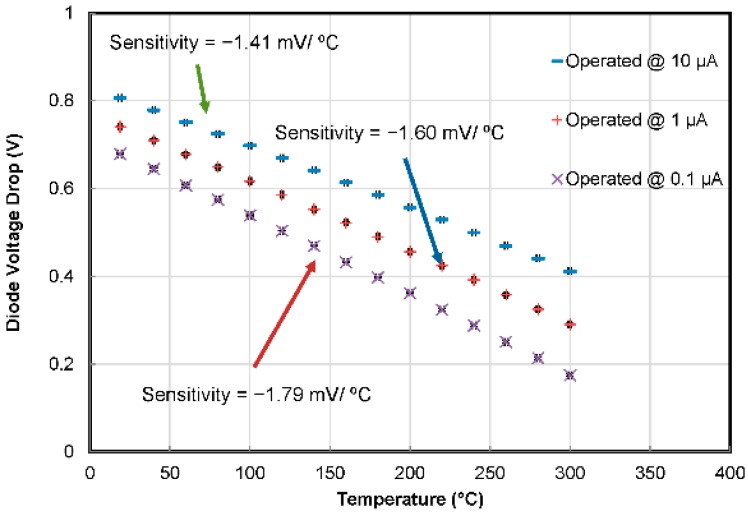
Calibration curve of two different thermodiodes from two different SOI CMOS MEMS chips operated at three different current levels with standard deviation error bars. The temperature sensors have shown an excellent repeatability of the results.

With increasing temperature, the *I-V* curves shift leftwards. We can see that at constant current, the diode voltage drops with increasing temperature. The voltage-temperature relation of two temperature sensors (TS2 and TS5) on different chips is plotted in Figure 11 for constant current levels of 10 µA, 1 µA and 0.1 µA. To avoid self-heating, low forward driving currents have been applied. The thermodiodes’ sensitivity was recorded to be −1.41 mV/°C, −1.60 mV/°C and −1.79 mV/°C for driving currents of 10 µA, 1 µA and 0.1 µA, respectively. It is worth mentioning that decreasing the drive current increases the sensitivity of the temperature sensors. The repeatability of the results was verified with the help of extensive tests, and the results demonstrate high inter-chip reproducibility of the thermodiodes’ characteristics/performance.

### 4.2. Pressure Sensor

**Figure 12:**
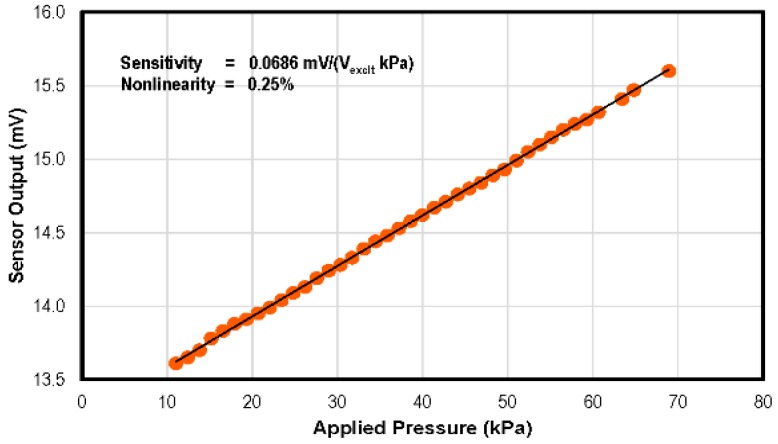
Response of the pressure sensor to the applied pressure. The sensor output voltage vs. applied pressure curve has 0.25% non-linearity against the best fit straight line.

**Figure 13:**
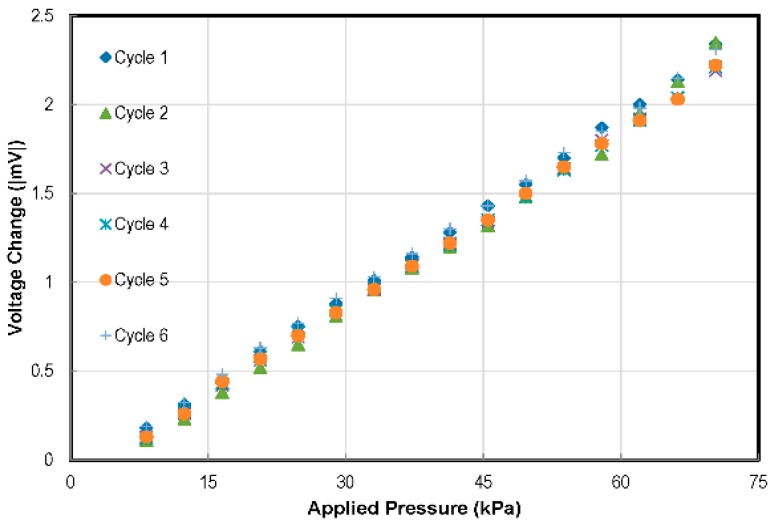
Repeatability test results of the pressure sensor in the SOI CMOS MEMS chip.

An excitation voltage (*V_excit_*) of 500 mV was provided to the pressure sensor. The calibration results, as shown in Figure 12, represent the sensitivity of 0.0686 mV/(V_excit_ kPa) and 0.25% nonlinearity. To avoid the use of complex compensation circuitry in the sensing systems, designers generally desire sensors to have a linear response. Capacitive pressure sensors have a very non-linear response as compared to the piezoresistive pressure sensors. The desired linearity of the piezoresistive sensors is evident in the results obtained. To evaluate sensor performance repeatability, a number of pressure cycles were applied, and the sensor response was noted. The results are plotted in Figure 13.

A number of factors influence the repeatability of piezoresistive pressure sensors. As reported in the literature, piezoresistors are known to be sensitive to temperature changes [34,35]. Temperature changes cause an offset in the calibration charts. Furthermore, due to the misalignment of piezoresistors on the sensor membrane, the response of the sensor may drift from the desired results. Similarly, temperature changes effect membrane deflection due to the expansion/contraction of trapped gasses under the sensor membrane. Detailed analyses of the factors influencing the repeatability of piezoresistive pressure sensors and measures to address them are being carried out by our group and will be reported separately.

### 4.3. Flow Rate Sensor

The flow in a closed channel has to be fully developed for the sensor to measure the flow rate effectively. A flow is said to have been fully developed when it acquires a steady velocity profile in a duct/closed channel. The flow rate sensor has been calibrated to estimate the laminar air flow rate in the designed micro-channel. In order to be sure that the laminar flow inside the duct/closed channel is fully developed, the sensor can only be placed beyond the entrance length (*Le*), which is given by following correlation [36]:

(7) Le ≈0.06 Re

Here, *Re* represent the Reynolds number of air flow in the micro-channel.

**Figure 14:**
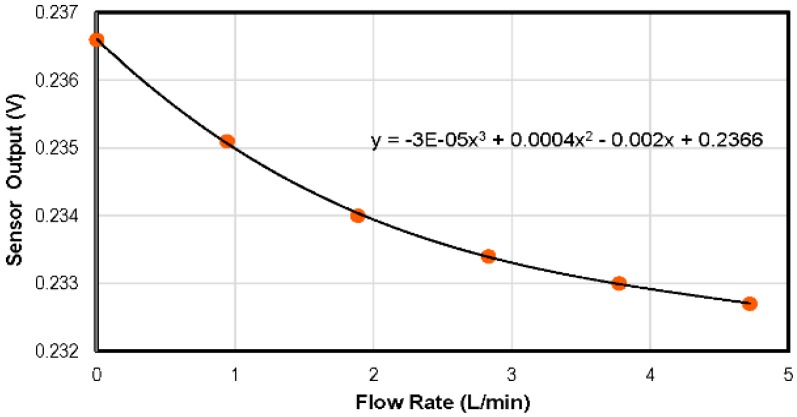
Response of the aluminium-based flow rate sensor in the multi-sensor SOI CMOS MEMS chip. A constant current of 40 mA was used to heat the embedded metal film.

**Figure 15:**
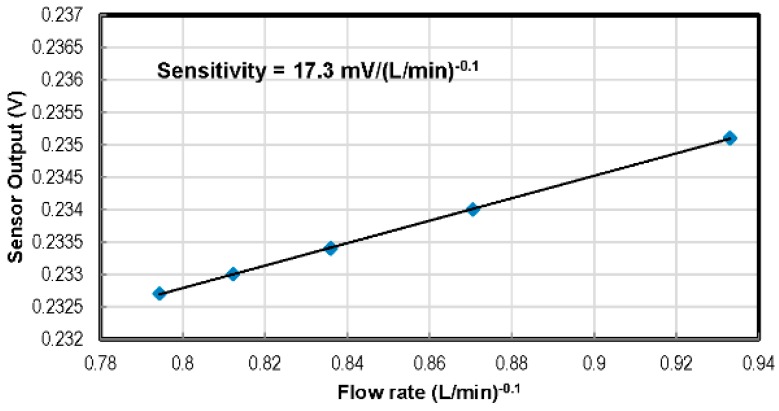
Linearized calibration curve of the aluminium hot film-based flow rate sensor operated in constant current mode at 40 mA.

To determine whether the flow in a duct is laminar or turbulent, one has to look at the value of the Reynolds number of the flow [37]. If the Reynolds number is lower than 2000, the flow is considered purely laminar. The sensor was placed 20 mm from the inlet, where the flow was calculated to be in the fully-developed laminar flow regime. Flow rate calibration was carried out using dry air with the hot film sensor operated in constant current mode at a 40-mA drive current. The third order polynomial calibration curves of the thermal flow sensor, which is quite similar to some reported in the literature [38,39], are depicted in Figure 14. Figure 15 presents the linearized calibration curve showing the sensitivity of 17.3 mV/(L/min)^−0.1^.

The performance comparison of our multi-sensor MEMS chip with some similar sensing systems reported in the literature is summarized in Table 2. It is worth mentioning that while our multi-sensor chip is capable of sensing these parameters with ranges and sensitivities comparable with the previously reported studies, it demonstrates the highest-ever sensor packing density. This has been possible due to the use of SOI and CMOS technologies in the fabrication processes, as well as the utilization of DRIE for membrane-based flow rate and pressure sensors’ fabrication.

## 5. Conclusions

The design, fabrication and characterization of a multi-sensor SOI CMOS MEMS chip that can simultaneously sense flow rate, pressure and temperature of a fluid have been successfully demonstrated. The use of SOI and CMOS technology in the fabrication process of the chip, as well as the application of the DRIE process for membrane formation, has resulted in a high packing density of the sensors on the chip (1.38 sensors per millimetre square of chip area).

The design has focused on sensing parameters that are of keen interest for scientists and researchers working in the field of experimental fluid dynamics. Independent calibration apparatuses have been used for the characterization of each type of sensor. The sensitivity of temperature sensors turned out to be −1.41 mV/°C to −1.79 mV/°C for a drive current of 10 µA–0.1 µA in the tested range of 20 °C–300 °C. Sensors from different chips were tested and found to have excellent reproducibility. In the range of 0–69 kPa above the ambient pressure, the pressure sensor showed a sensitivity of 0.0686 mV/(V_excit_ kPa) with 0.25% non-linearity. A micro-channel was used for the characterization of the flow rate sensor, which showed a linearized sensitivity of 17.3 mV/(L/min)^−0.1^. The chip can potentially be used in fluidic experiments for real-time flow parameters’ monitoring in wind tunnels and automotive, aerospace, biomedical, microfluidic systems/applications.

## Figures and Tables

**Table 1:** Summary of the key multi-sensor MEMS chips reported in the literature since 1987.

S No	Year	Ref.	No of Sensors	Parameters Sensed													Substrate		CMOS Process	
				Flow Rate/Mass Flow	Wall Shear Stress	Pressure	Temperature	Gas	Density	Light Intensity	Electric Conductivity	Flow Direction	Humidity	Air Speed	Magnetic Field	Acceleration	Silicon	SOI	Yes	No
1	1987	[9]	3	√		√	√										√			√
2	1992	[15]	5			√	√	√				√		√			√		√	
3	1996	[10]	3		√	√	√											√		√
4	2000	[2]	4	√	√	√	√										√			√
5	2001	[18]	4				√	√									√		√	
6	2002	[3]	4				√	√									√		√	
7	2004	[4]	3	√			√		√								√			√
8	2005	[11]	4			√	√			√	√						√			√
9	2005	[5]	3		√	√	√										√			√
10	2005	[17]	3			√	√						√				√ ^1^			√ ^2^
11	2007	[16]	4			√	√						√			√		√		√ ^2^
12	2009	[6]	3	√			√					√					√			√
13	2011	[8]	2	√		√											√		√	
14	2013	[7]	8			√	√			√		√	√	√	√	√		√		√

^1^ Sensor chips have been fabricated using silicon wafers bonded with glass wafers; ^2^ non-CMOS Silicon/SOI sensor chips have been packaged with independent Bi-CMOS interface circuitry chips.

**Table 2:** Performance comparison of the selected multi-sensor MEMS chips capable of measuring pressure, temperature and flow rate.

Reference	Year	Parameter						Chip Dimensions (mm × mm)	No. of Sensors on the Chip	Sensor Density (Number of Sensors Per mm^2^ of Chip Area)
		Pressure		Temperature		Flow Rate				
		Range (kPa)	Sensitivity |µV/(V·kPa)|	Range (°C)	Sensitivity	Range (L/min)	Sensitivity (V/(L/min))			
[9]	1987	0–40	9.9 at 1 mA	25–45	N/R ^1^	0–7	N/R	4 × 8	3	0.1875
[15]	1992	0–107	92.3	−55 to 125	4 mV/°C	N/R	N/R	3.5 × 5	6	0.3429
[5]	2005	0–140	82.9	30–90	TCR ^2^ 0.104% °C^−1^	0–5	0.17 to 0.62	20 × 10	15	0.0750
[17]	2005	67–160	113 fF/kPa	20–100	15 fF/°C	N/R	N/R	~3 × 4	5	0.4167
[8]	2011	0–50	3900 @ 21 gain	N/R	N/R	0–5	0.01 to 0.09	4.5 × 9	2	0.0494
[7]	2013	67–100	16,000 @ 500 gain	30–50	0.72–2.9 mV/°C	N/R	N/R	10 × 10	12	0.1200
This work	2016	0–75	69	**20–300**	−1.41 to −1.79 mV/°C	0–4.7	−0.32 to −1.59	3.8 × 3.8	**20**	**1.3800**

^1^ N/R = Not Reported; ^2^ Temperature Coefficient of Resistance.
